# Liver kinase B1 enhances chemoresistance to gemcitabine in breast cancer MDA-MB-231 cells

**DOI:** 10.3892/ol.2014.2446

**Published:** 2014-08-14

**Authors:** CHEN XIA, FUGUI YE, XIN HU, ZHENGDONG LI, BEIQI JIANG, YUN FU, XIAOLIN CHENG, ZHIMING SHAO, ZHIGANG ZHUANG

**Affiliations:** 1 Department of Breast Surgery, Shanghai First Maternity and Infant Hospital, Tongji University, School of Medicine, Shanghai 200040, P.R. China; 2 Department of General Surgery, Affiliated Union Hospital of Fujian Medical University, Union Clinical School, Fujian Medical University, Fuzhou, Fujian 350001, P.R. China; 3 Department of Breast Surgery, Cancer Center and Cancer Institute, Shanghai Medical College, Fudan University, Shanghai 200032, P.R. China

**Keywords:** breast neoplasms, cytidine deaminase, DNA damage, gemcitabine, liver kinase B1

## Abstract

Liver kinase B1 (LKB1) is a well-known tumor suppressor gene in a variety of human cancers, including breast cancer. However, its role in gemcitabine resistance is unclear. Since gemcitabine in combination with other chemotherapeutic reagents is the first-line treatment in advanced breast cancer, the aim of the present study was to determine the effect of ectopic expression of LKB1 on chemosensitivity to gemcitabine in the breast cancer MDA-MB-231 cell line. Increasing the expression of LKB1 was found to directly correlate with gemcitabine chemoresistance. Although LKB1 suppressed the cell proliferation rate and clonogenicity in the absence of gemcitabine, it increased the median inhibitory concentration of gemcitabine and clonogenicity of cells in the presence of gemcitabine. Mechanistic analysis indicated that LKB1 was able to protect cells from DNA damage caused by gemcitabine. Furthermore, it was found that LKB1 induced a significant upregulation of cytidine deaminase expression, an important enzyme that accelerates gemcitabine catabolization. Overall, dual characteristics of LKB1 were identified: Suppressing cell growth in normal conditions and enhancing chemoresisitance to gemcitabine, possibly by accelerating degradation of gemcitabine, and protecting cells from DNA damage caused by gemcitabine.

## Introduction

The liver kinase B1 (LKB1)/serine-threonine kinase 11 tumor suppressor gene encodes a ubiquitously expressed and evolutionarily conserved serine-threonine kinase. LKB1 was originally identified as a tumor suppressor gene due to its association with an increased risk of malignancy and Peutz-Jeghers syndrome (PJS; a rare autosomal dominant syndrome characterized by benign polyps of the gastrointestinal tract) (1). An increased incidence of carcinomas of the gastrointestinal tract, as well as breast, ovarian, uterine, cervical, lung and testicular cancer, has been observed in PJS patients and their relatives (2,3). Somatic mutations in LKB1 have also been observed in sporadic pulmonary, breast, pancreatic and biliary cancers and melanomas (4–8). Although LKB1 has been identified as a tumor suppressor, its function in chemoresistance remains unclear.

Gemcitabine, also known as 2′,2′-difluorodeoxycytidine (dFdC) or Gemzar, is an analogue of deoxycytidine, with two fluorine atoms substituted at the 2′-position of the ribose ring. It has been widely used in the treatment of several types of cancer, including non-small cell lung, pancreatic and metastatic breast cancer (9–11). After entering the cell, gemcitabine is phosphorylated to gemcitabine monophosphate (dFdCMP), diphosphate (dFdCDP) and triphosphate (dFdCTP) in a stepwise manner (12,13). dFdCTP may be incorporated into DNA, leading to strand termination and cellular apoptosis. Other mechanisms associated with the anticancer effects of gemcitabine include ribonucleotide reductase (RNR) inhibition, RNA incorporation and thymidylate synthase inhibition (12). However, one of the main factors hindering gemcitabine application is its chemoresistance. Resistance to gemcitabine may be attributed to cellular events during drug uptake and metabolism, and a number of molecular markers have been found to correlate with gemcitabine sensitivity (14).

To the best of our knowledge, the present study is the first to investigate the effect of forced LKB1 expression on chemosensitivity to gemcitabine in breast cancer cells.

## Materials and methods

### Cell lines and cell culture

The human breast cancer cell line, MDA-MB-231, was obtained from the American Type Culture Collection (Manassas, VA, USA). The MDA-MB-231 gemcitabine resistance subline was developed by over one year of exposure to gemcitabine, beginning with 1 μM and increasing stepwise to 840 μM. Cells were maintained in Leibovitz’s L-15 medium (Sigma-Aldrich, St. Louis, MO, USA) containing 10% fetal bovine serum (Gibco-BRL, Carlsbad, CA, USA), 100 units/ml penicillin and 100 μg/ml streptomycin (Gibco-BRL) at 37°C in a humidified atmosphere with 5% CO_2_.

### Adenovirus production and transfection

The coding sequence of LKB1 was amplified by polymerase chain reaction (PCR) and then cloned into the adenoviral vector plasmid with the flag tag, using the Gateway Cloning system (Invitrogen Life Technologies, Carlsbad, CA, USA). The LKB1 expression plasmids and corresponding vector plasmids were transfected into HEK-293T cells with the gag-pol packaging and VSV-G envelope plasmids using polyethylenimine reagent. The medium containing virus particles was harvested. Cells were seeded into a 25-cm^2^ cell culture flask 1 day prior to transfection, and 2 ml of collected medium containing virus particles, 2 ml L15 medium and 4 μl 8 mg/ml polybrene mixture was added to transfect the cells. Medium containing 1 μg/ml puromycin was used to screen for stable transfected cells.

### RNA isolation, reverse transcription and quantitative PCR (qPCR)

Total RNA was extracted from cells with TRIzol reagent (Invitrogen Life Technologies, Carlsbad, CA, USA) and the reverse transcription reaction was performed using the Reverse Transcription system (Promega Corporation, Shanghai, China). qPCR was performed using SYBR Premix Ex Taq (Takara Biotechnology (Dalian) Co., Ltd., Dalian, China), and GAPDH was used as an internal control. The primer sequences used were as follows: Forward, 5′-GAGAAGCGTTTCCCAGTGTG-3′ and reverse, 5′-CCCAGGTCGGAGATTTTGA-3′ for LKB1; and forward, 5′-GGATTTGGTCGTATTGGG-3′ and reverse, 5′-GGATTTGGTCGTATTGGG-3′ for GAPDH.

### Western blot analysis

Cells were washed twice with phosphate-buffered saline (PBS) followed by T-PER tissue extraction buffer (Thermo Fisher Scientific, Rockford, IL, USA) supplemented with protease and phosphatase inhibitor tablets (Roche Diagnostics, Indianapolis, IN, USA) on ice for 30 min. The lysates were then centrifuged at 12,000 g for 30 min at 4°C. Next, a total of 20 μg protein was resolved by sodium dodecyl sulfate-polyacrylamide gel electrophoresis and transferred to polyvinylidene fluoride film. The film was then incubated with blocking solution containing 5% bovine serum albumin [BSA; Sangon Biotech (Shanghai) Co., Ltd., Shanghai, China) in Tris-buffered saline with Tween 20 [Sangon Biotech (Shanghai) Co., Ltd.] at room temperature for 1 h. Subsequently, the film was immunoblotted with polyclonal rabbit anti-human LKB1 (Calbiochem, Dormstadt, Gemany), monoclonal mouse anti-human ribonucleotide reductase M1 (RRM1; Abcam, Hong Kong, China), monoclonal rabbit anti-human phosphorylated (p)-ATR, monoclonal rabbit anti-human p-CHK1, monoclonal rabbit anti-human p-ATR, monoclonal rabbit anti-human p-CHK2 and monoclonal mouse anti-human γH2AX (Cell Signaling Technology, Inc., Danvers, MA, USA) antibodies, or monoclonal mouse anti-human GAPDH antibody (Sigma-Aldrich). Polyclonal goat anti-rabbit and goat anti-mouse IgG (H+L) (Jackson ImmunoResearch Laboratories, Inc., West Grove, MA, USA) secondary antibodies were used. The signals were detected using chemiluminescent horseradish peroxidase substrate (Millipore, Billerica, MA, USA) according to the manufacturer’s instructions.

### Cytotoxicity and cell proliferation assays

Cells at the logarithmic growth phase were plated at a density of 2×10^3^ cells/well in 96-well plates. Following overnight adherence, complete medium was replaced with medium containing 16 different concentrations of gemcitabine ranging between 0.00001 and 900 μmol/l. Following gemcitabine treatment for six days, cell cytotoxicity was measured by the Cell Counting Kit-8 (CCK-8) kit (Dojindo Laboratories, Kumamoto, Japan). The IC_50_ value of gemcitabine was estimated from semilogarithmic dose-response curves generated using GraphPad Prism (GraphPad Software, Inc., La Jolla, CA, USA). Each experiment was performed in triplicate. Cell proliferation rate was also measured using the CCK-8 kit every 24 hours for seven days and a proliferation curve was generated using Prism 5 software (GraphPad Software, Inc.).

### Colony formation assay

The cells were seeded at a density of 500 cells per 60-mm dish. Following 24 h, complete medium was replaced with medium containing different concentrations of gemcitabine (0.0001, 0.0003 and 0.0006 μmol/l). In addition, 100 cells of each type were seeded as standard controls, without gemcitabine treatment. After 14 days, clones were fixed and stained with crystal violet containing 40% methanol for ~30 min. Stained clones with a diameter of >1 mm were counted and standardized. The cloning efficiency was calculated using the following formula: Cloning efficiency (%) = (clone number/total cell number)/(control clone number/control total cell number) × 100. Each independent experiment was performed in triplicate. For colony formation assays without gemcitabine treatment, 300 cells were seeded in each dish.

### Immunofluorescence assay

Cells were seeded at a density of 2×10^4^ cells/well onto coverslips in a 24-well plate. Following 30 min of fixation in 4% paraformaldehyde, 10 min of permeabilization with 0.5% Triton X-100 solution and 1 h of blockage with 5% BSA in PBS, cells were incubated with primary monoclonal mouse anti-human γH2AX antibody (Millipore) for 2 h, and Alexa Fluor 555 polyclonal goat anti-rabbit IgG (H+L) secondary antibody (red; Invitrogen Life Technologies) for 1 h. DAPI was used to stain the nuclei. All images were captured using a confocal laser microscope (Leica TCS SP5; Leica, Mannheim, Germany).

### Statistical analysis

Statistical analysis was performed using SPSS software, version 17.0 (SPSS, Inc., Chicago, IL, USA). One-way analysis of variance was used to determine the statistical significance of differences between experimental groups. P<0.05 was considered to indicate a statistically significant difference.

## Results

### LKB1 suppresses the proliferation of breast cancer cells

**Figure 1:**
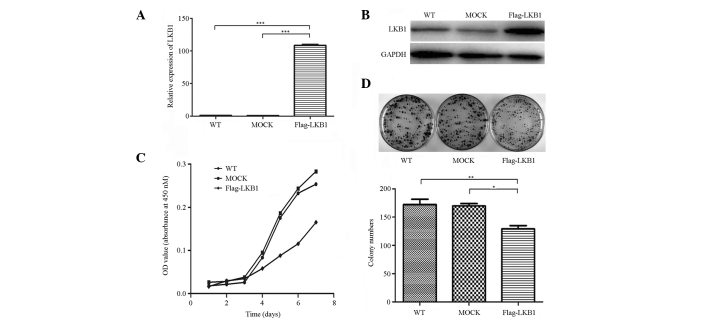
LKB1 suppresses the proliferation of breast cancer cells. (A) Relative expression of LKB1 in cells measured by quantitative polymerase chain reaction. Each experiment was performed in triplicate and data are presented as the mean ± SD. (B) Expression of LKB1 in cells was detected by western blot analysis. (C) Cell proliferation assays revealed that LKB1 increased the cell proliferation rate. (D) Colony formation assays revealed weaker clonogenicity in LKB1 transfected cells when compared with wild-type and mock-transfected cells. (^*^P<0.05, ^**^P<0.01 and ^***^P<0.001). LKB1, liver kinase B1; WT, wild-type; OD, optical density.

Successful construction of LKB1 stably transfected cells and the mock-transfected controls was determined by qPCR, and further confirmed by western blot analysis (Fig. 1A and B). To investigate the function of LKB1 in breast cancer cells, cell proliferation and colony formation assays were performed. The results indicated that LKB1 markedly decreased cell proliferation rate and clonogenicity (Fig. 1C and D). These results were consistent with previous studies, which have identified LKB1 as a tumor suppressor gene (15,16).

### Forced expression of LKB1 is associated with increased gemcitabine chemoresistance

**Figure 2:**
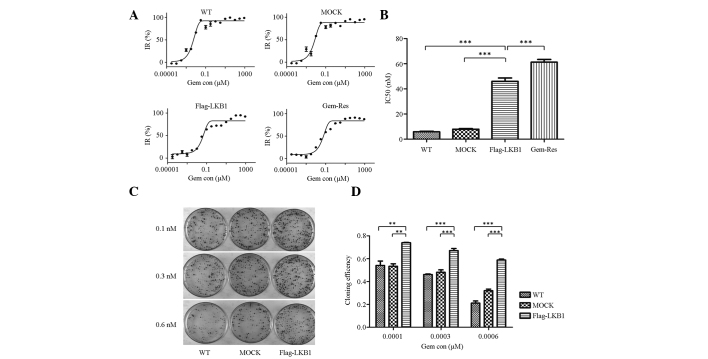
Forced LKB1 expression is associated with increased gemcitabine chemoresistance. (A) Semilogarithmic dose-response curves of cytotoxicity assays as measured by Cell Counting Kit-8 assay. (B) Estimated IC_50_ value of wild-type, mock-transfected, LKB1-transfected and gemcitabine-resistant sublines of MDA-MB-231 cells. The IC_50_ value of LKB1-transfected cells was higher than that of wild-type and mock-transfected cells; however, it was lower than that of the gemcitabine-resistant subline. Each independent experiment was performed in triplicate and data are presented as the mean ± SD. (C) Cloning efficiency of the three cell lines at three different concentrations (0.1, 0.3 and 0.6 nM) of gemcitabine. The control group contained 100 cells/dish without gemcitabine. Cloning efficiency was calculated using the following formula: Cloning efficiency (%) = (clone number/total cell number)/(control clone number/control total cell number) × 100. Each independent experiment was performed in triplicate and the data are presented as the mean ±SD. (D) Colony formation assays revealed higher clonogenicity in LKB1-transfected cells when compared with wild-type and mock-transfected cells, in the presence of gemcitabine. (^*^P<0.05, ^**^P<0.01 and ^***^P< 0.001). IR, inhibition rate; Gem con, gemcitabine concentration; LKB1, liver kinase B1; WT, wild type.

To further explore the effect of LKB1 on gemcitabine sensitivity in breast cancer cells, a cytotoxicity assay was conducted. Semilogarithmic dose-response curves from the cytotoxicity assays of wild-type, mock-transfected, LKB1-transfected and gemcitabine-resistant MDA-MB-231 cells are shown in Fig. 2A. In addition, the estimated IC_50_ values of these cells are shown in Fig. 2B. The IC_50_ value of LKB1-transfected cells was 46.07 nM, which was more than seven-fold greater that of MDA-MB-231 cells (5.85 nM) (P<0.001), more than five-fold greater than that of mock-transfected cells (7.93 nM) (P<0.001), and less than that of gemcitabine-resistant cells (61.27 nM) (P<0.001).

In the colony formation assay, the colony number decreased with increasing gemcitabine concentration in each cell line. Furthermore, in each gemcitabine concentration group, LKB1 increased the colony number when compared with wild-type (P<0.01) or mock-transfected (P<0.01) cells (Fig. 2C and D). Cloning efficiency was standardized by control group (100 cells/dish, without gemcitabine) to exclude the inaccuracy of manual operation and culturing conditions. Overall, the cytotoxicity and colony formation assays indicated that LKB1 enhances chemoresistance to gemcitabine.

### LKB1 overexpression alleviates gemcitabine-induced DNA damage

**Figure 3:**
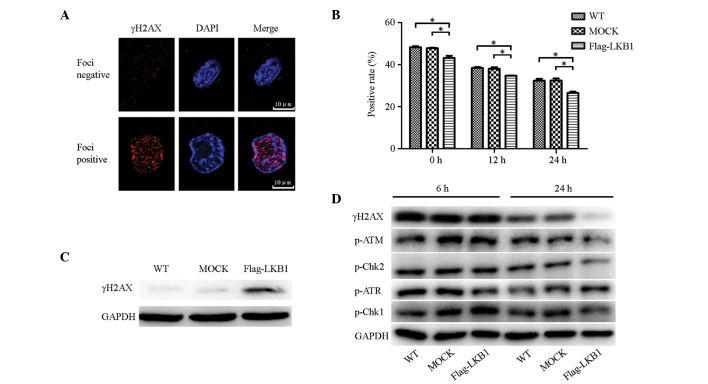
LKB1 overexpression alleviates gemcitabine-induced DNA damage possibly by affecting the ATM-CHK2 pathway. (A) Negative and positive examples of γH2AX foci in the immunofluorescence assay. (B) Positive rates of γH2AX foci in cells. Following gemcitabine treatment (1 μM; 24 h), three time points (0, 12 and 24 h) were selected to perform the immunofluorescence assay. Three fields were randomly selected for each coverslip and positive rates were calculated. LKB1 decreased positive rates of γH2AX foci. Each independent experiment was performed three times and data are presented as the mean ± SD. (C) γH2AX expression in normal conditions was analyzed by western blot analysis. LKB1 increased γH2AX expression. (D) Expression of DNA damage-associated proteins (p-ATR, -CHK1, -ATR, -CHK2 and γH2AX) 6 and 24 h following gemcitabine treatment (1 μM; 24 h), respectively. LKB1 affected the expression of p-ATR, p-CHK2 and γH2AX in the 24 h group. (^*^P< 0.05, ^**^P<0.01 and ^***^P<0.001). LKB1, liver kinase B1; p, phosphorylated; WT, wild-type.

γH2AX expression was analyzed using immunofluorescence to assess the effect of LKB1 on the DNA damage caused by gemcitabine. Cells were fixed at various time points (0, 12 and 24 h) following treatment with 1 μM gemcitabine for 24 h, followed by an immunofluorescence assay to detect γH2AX foci, the marker of DNA double-strand breaks (DSBs), in cell nuclei. Cells with clear red foci in the nucleus were considered to be DNA damage-positive, while those without were considered as negative (Fig. 3A). Three fields were randomly selected for each coverslip to calculate positive rates (Fig. 3B). In each cell line, positive rates decreased in a time-dependent manner. At each time point, the positive rate for LKB1-transfected cells was lower than that for wild-type and mock-transfected cells, and the differences were statistically significant.

In addition, western blot analysis was used to compare γH2AX expression levels prior to and following treatment with 1 μM gemcitabine for 24 h. In the absence of gemcitabine, low levels of γH2AX were expressed in wild-type and mock-transfected cells, while LKB1-transfected cells expressed significantly more γH2AX (Fig. 3C). Notably, this trend was gradually reversed following gemcitabine treatment and, 6 h following gemcitabine withdrawal, γH2AX expression in the wild-type and mock-transfected cells increased to the same level as that in the LKB1-transfected cells. Furthermore, 24 h following gemcitabine withdrawal, as DNA repair progressed, γH2AX expression began to decline, decreasing most significantly in LKB1-transfected cells (Fig. 3D). This result indicated that LKB1 expression activates a process of resistance to the DNA damage caused by gemcitabine. This protective function was not evident directly following exposure to gemcitabine; however, it was identified several hours following the withdrawal of gemcitabine treatment.

In addition to γH2AX, other proteins associated with DNA damage (p-ATR, -CHK1, -ATM and -CHK2) were examined by western blot analysis following gemcitabine treatment (1 μM for 24 h). The protein expression 24 h following gemcitabine withdrawal was generally lower than that of the 6-h group, indicating that less DNA damage had occurred with time. In the 6-h group, p-ATM and -CHK2 expression was almost equivalent in the three cell lines; whereas in the 24 h group, the expression was markedly lower in the LKB1-transfected cells, exhibiting the same trend as γH2AX. However, p-ATR and -CHK1 did not exhibit a similar trend (Fig. 3D). This indicated that LKB1 only regulates the ATM-CHK2 pathway, and not the ATR-CHK1 pathway, in response to gemcitabine.

### Ectopic expression of LKB1 increases the expression of cytidine deaminase (CDA)

**Figure 4:**
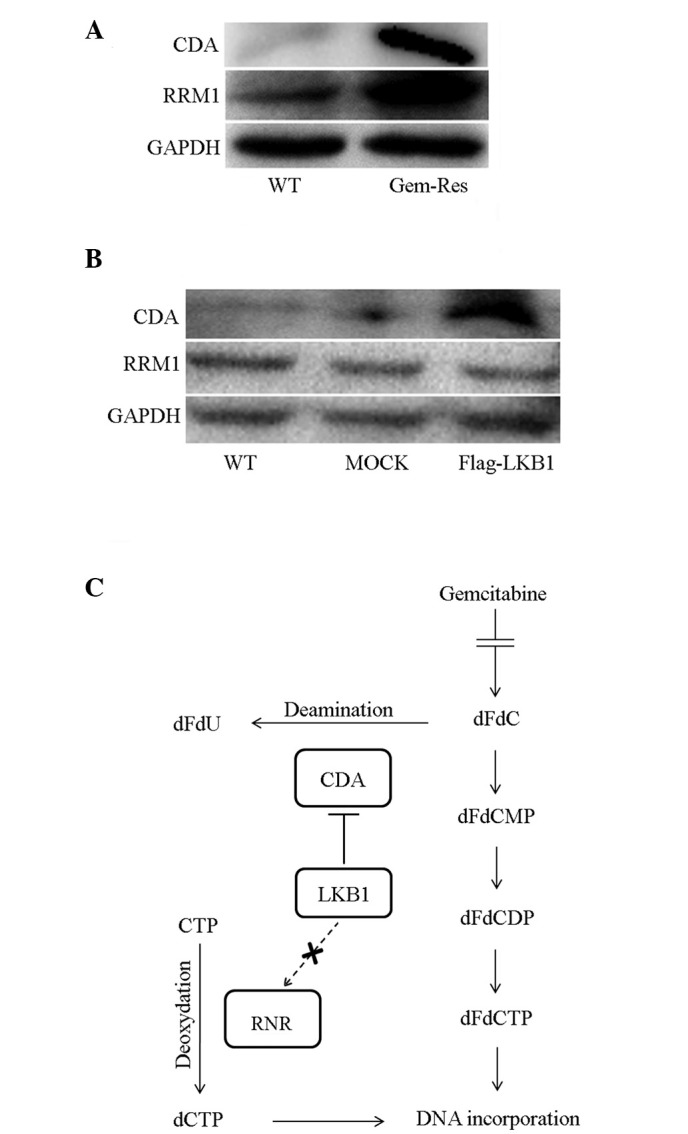
Ectopic expression of LKB1 increases the expression of CDA. (A) CDA and RRM1 expression in wild-type and gemcitabine-resistant MDA-MB-231 cells was analyzed by western blot analysis. The two enzymes were upregulated in the gemcitabine-resistant subline. (B) Western blot analysis of CDA and RRM1 expression in cells. LKB1 increased CDA expression. (C) Metabolism and mechanism of gemcitabine in the cytoplasm. CDA is the enzyme that deaminizes gemcitabine to dFdU, eliminating the anticancer effect of gemcitabine. RRM1 is a subunit of RNR, which is the key enzyme involved in the production of the deoxyribonucleotide pool and DNA synthesis (12). LKB1, liver kinase B1; CDA, cytidine deaminase; RNR, ribonucleotide reductase; RRM1, ribonucleoside-diphosphate reductase; dFdCMP, difluoro-deoxyuridine monophosphate; dFdCDP, difluoro-deoxyuridine diphosphate; dFdCTP, difluoro-deoxyuridine triphosphate; WT, wild type.

Following on from the previous results, the mechanism whereby LKB1 enhances gemcitabine resistance was further investigated. CDA is an enzyme that catabolizes gemcitabine to dFdU, thus abolishing the cytotoxicity of gemcitabine. RRM1 is a component of RNR, which antagonizes the DNA-damaging effect of gemcitabine. The expression of the two enzymes was detected by western blot analysis in different cells. As shown in Fig. 4A, CDA and RRM1 expression was higher in gemcitabine-resistant sublines than in wild-type MDA-MB-231 cells. However, only CDA expression was upregulated in LKB1-transfected cells (Fig. 4B). Therefore, we hypothesized that the upregulation of CDA expression is one mechanism by which LKB1 reduces sensitivity to gemcitabine in breast cancer cells.

## Discussion

LKB1 has been identified as a tumor suppressor gene in numerous studies (15); however, its function in chemoresistance remains unclear. To the best of our knowledge, the present study was the first to identify that LKB1 enhances gemcitabine resistance in the breast cancer MDA-MB-231 cell line, possibly by accelerating gemcitabine degradation and protecting cells from DNA damage.

The cytotoxicity and colony formation assays supplemented with gemcitabine revealed that LKB1-transfected cells grew faster and formed more colonies than wild-type and mock-transfected MDA-MB-231 cells, indicating that LKB1 enhanced the chemosensitivity to gemcitabine. Notably, in the proliferation and colony formation assays without gemcitabine treatment, LKB1 suppressed cell proliferation and reduced cell clonogenicity, which was consistent with the results of previous studies (16). Overall, the protective role of LKB1 in breast cancer cells was specific to the gemcitabine environment.

To further investigate the association between LKB1 and gemcitabine, and as gemcitabine inhibited DNA synthesis and caused DNA damage, the extent of DNA damage was evaluated in LKB1-transfected, mock-transfected and wild-type MDA-MB-231 cells following exposure to gemcitabine. γH2AX is a generally accepted sensitive marker of DNA damage, and in particular DSBs. γH2AX is the phosphorylated form of H2AX, a member of the H2A family. The H2A family is a histone family that facilitate the organization of chromatin. The detection of γH2AX by immunofluorescence is a widely used and convenient method of assessing DNA damage, which enables the visualization of the γH2AX foci, and the quantification of γH2AX foci indicates the degree of DNA damage. A number of genotoxic insults may lead to DSBs, including ionizing radiation, ultraviolet light exposure, drugs and chemicals, among others, of which gemcitabine is one (17,18). Previous studies have shown that gemcitabine treatment may induce γH2AX foci in the nucleus, indicating damage to the DNA (19,20). In the present study, according to the immunofluorescence assay and calculated foci positive rates, in each cell line, foci positive rates generally decreased in a time-dependent manner following gemcitabine withdrawal. This may have been a result of gemcitabine consumption or DNA repair. At each time period, LKB1 transfected cells exhibited a lower foci positive rate than wild-type or mock-transfected cells (P<0.01).

Additionally, γH2AX expression was investigated by western blot analysis prior to and following gemcitabine treatment. On the one hand, γH2AX expression was the highest in LKB1-transfected cells in the absence of gemcitabine; however, on the other hand, it was the lowest following exposure to gemcitabine. Therefore, we hypothesized that LKB1 acts as a tumor suppressor by increasing cell vulnerability to DSBs in the normal environment; however, in the presence of gemcitabine, other mechanisms were activated in LKB1-transfected cells to eliminate this vulnerability to DSBs. Overall, we hypothesized that LKB1 prevented breast cancer cells from DSBs caused by gemcitabine, and enhanced the chemoresistance to gemcitabine.

In addition to γH2AX, other proteins involved in DNA damage pathways (p-ATR, -CHK1, -ATM and -CHK2) were examined following exposure to gemcitabine. The ATR-CHK1 and ATM-CHK2 protein kinase pathways are the two predominant signaling pathways activated by DNA damage (21). ATM is activated primarily by radiation and genotoxins that induce DNA DSBs (22), while ATR is activated via recruitment to the single-stranded DNA (23). The activation of ATM at DSB sites may affect multiple local substrates, including CHK2 and H2AX, inducing their phosphorylation (24,25). As shown in Fig. 3D, among p-ATR, -CHK1, -ATM and -CHK2, only p-ATM and -CHK2 exhibited the same trend as γH2AX, supporting the hypothesis that LKB1 prevents DSB caused by gemcitabine via the ATM-CHK2 pathway.

The enhancement of resistance to gemcitabine is possibly due to the involvement of LKB1 in gemcitabine metabolism; therefore, the expression of CDA and RRM1 in wild-type and transfected cells was assessed. The metabolism and mechanism of gemcitabine following entrance to the cytoplasm is shown in Fig. 4C. CDA is the enzyme that catabolizes gemcitabine to dFdU, preventing the stepwise conversion of gemcitabine into dFdCMP, dFdCDP and dFdCTP and inhibiting DNA synthesis. Previous study has revealed the function of CDA in gemcitabine resistance (26). Another important enzyme involved in gemcitabine metabolism is RNR. It converts ribonucleotide 5′-diphosphates to 2′-deoxyribonucleotide-5′-diphosphates, which is the rate limiting step for deoxyribonucelotide production and DNA synthesis. RRM1 is a subunit of RNR, which has been reported to contribute to gemcitabine chemoresistance *in vivo* and *in vitro* (27,28). In the present study, CDA levels were highest in LKB1-transfected cells; however, no significant differences in RRM1 levels were identified between wild-type, mock-transfected and LKB1-transfected cells. The higher levels of CDA were partly responsible for the decreased number of DSBs and increased resistance to gemcitabine in LKB1-transfected cells. However, other mechanisms may account for LKB1 enhancing gemcitabine resistance.

To the best of our knowledge, the current study is the first to indicate that LKB1 is a tumor suppressor and gemcitabine desensitizer, simultaneously. As a result, when patients exhibit LKB1 expression in breast cancer, the application of gemcitabine may not achieve the expected outcome. Since LKB1 is a potential treatment target for malignant tumors, its ability to enhance chemoresistance to gemcitabine must be considered during subsequent oncological management. In conclusion, the results of the current study provide a novel insight into the antitumor activity of gemcitabine and indicate a distinct mechanism for improving the efficacy of gemcitabine, which is feasible for clinical application in breast cancer patients.

## References

[1] Mehenni H, Gehrig C, Nezu J (1998). Loss of LKB1 kinase activity in Peutz-Jeghers syndrome, and evidence for allelic and locus heterogeneity. Am J Hum Genet.

[2] Lim W, Olschwang S, Keller JJ (2004). Relative frequency and morphology of cancers in STK11 mutation carriers. Gastroenterology.

[3] Hearle N, Schumacher V, Menko FH (2006). Frequency and spectrum of cancers in the Peutz-Jeghers syndrome. Clin Cancer Res.

[4] Fenton H, Carlile B, Montgomery EA (2006). LKB1 protein expression in human breast cancer. Appl Immunohistochem Mol Morphol.

[5] Ji H, Ramsey MR, Hayes DN (2007). LKB1 modulates lung cancer differentiation and metastasis. Nature.

[6] Sanchez-Cespedes M, Parrella P, Esteller M (2002). Inactivation of LKB1/STK11 is a common event in adenocarcinomas of the lung. Cancer Res.

[7] Wang ZJ, Churchman M, Campbell IG (1999). Allele loss and mutation screen at the Peutz-Jeghers (LKB1) locus (19p13.3) in sporadic ovarian tumours. Br J Cancer.

[8] Ylikorkala A, Avizienyte E, Tomlinson IP (1999). Mutations and impaired function of LKB1 in familial and non-familial Peutz-Jeghers syndrome and a sporadic testicular cancer. Hum Mol Genet.

[9] Reck M, von Pawel J, Zatloukal P (2009). Phase III trial of cisplatin plus gemcitabine with either placebo or bevacizumab as first-line therapy for nonsquamous non-small-cell lung cancer: AVAil. J Clin Oncol.

[10] Cunningham D, Chau I, Stocken DD (2009). Phase III randomized comparison of gemcitabine versus gemcitabine plus capecitabine in patients with advanced pancreatic cancer. J Clin Oncol.

[11] Chan S, Romieu G, Huober J (2009). Phase III study of gemcitabine plus docetaxel compared with capecitabine plus docetaxel for anthracycline-pretreated patients with metastatic breast cancer. J Clin Oncol.

[12] Mini E, Nobili S, Caciagli B, Landini I, Mazzei T (2006). Cellular pharmacology of gemcitabine. Ann Oncol.

[13] Wong A, Soo RA, Yong WP, Innocenti F (2009). Clinical pharmacology and pharmacogenetics of gemcitabine. Drug Metab Rev.

[14] Bergman AM, Pinedo HM, Peters GJ (2002). Determinants of resistance to 2′,2′-difluorodeoxycytidine (gemcitabine). Drug Resist Update.

[15] Katajisto P, Vallenius T, Vaahtomeri K (2007). The LKB1 tumor suppressor kinase in human disease. Biochim Biophys Acta.

[16] Zhuang ZG, Di GH, Shen ZZ, Ding J, Shao ZM (2006). Enhanced expression of LKB1 in breast cancer cells attenuates angiogenesis, invasion, and metastatic potential. Mol Cancer Res.

[17] Bonner WM, Redon CE, Dickey JS (2008). GammaH2AX and cancer. Nature Rev Cancer.

[18] Mah LJ, El-Osta A, Karagiannis TC (2010). gammaH2AX: a sensitive molecular marker of DNA damage and repair. Leukemia.

[19] Al-Ejeh F, Darby JM, Brown MP (2007). The La autoantigen is a malignancy-associated cell death target that is induced by DNA-damaging drugs. Clin Cancer Res.

[20] Dufau I, Frongia C, Sicard F (2012). Multicellular tumor spheroid model to evaluate spatio-temporal dynamics effect of chemotherapeutics: application to the gemcitabine/CHK1 inhibitor combination in pancreatic cancer. BMC Cancer.

[21] Sancar A, Lindsey-Boltz LA, Unsal-Kaçmaz K, Linn S (2004). Molecular mechanisms of mammalian DNA repair and the DNA damage checkpoints. Ann Rev Biochem.

[22] Lee JH, Paull TT (2005). ATM activation by DNA double-strand breaks through the Mre11-Rad50-Nbs1 complex. Science.

[23] Zou L, Elledge SJ (2003). Sensing DNA damage through ATRIP recognition of RPA-ssDNA complexes. Science.

[24] Smith J, Tho LM, Xu N, Gillespie DA (2010). The ATM-Chk2 and ATR-Chk1 pathways in DNA damage signaling and cancer. Adv Cancer Res.

[25] Lee JH, Paull TT (2004). Direct activation of the ATM protein kinase by the Mre11/Rad50/Nbs1 complex. Science.

[26] Bardenheuer W, Lehmberg K, Rattmann I (2005). Resistance to cytarabine and gemcitabine and in vitro selection of transduced cells after retroviral expression of cytidine deaminase in human hematopoietic progenitor cells. Leukemia.

[27] Bergman AM, Eijk PP, Ruiz van Haperen VW (2005). In vivo induction of resistance to gemcitabine results in increased expression of ribonucleotide reductase subunit M1 as the major determinant. Cancer Res.

[28] Nakahira S, Nakamori S, Tsujie M (2007). Involvement of ribonucleotide reductase M1 subunit overexpression in gemcitabine resistance of human pancreatic cancer. Int J Cancer.

